# Towards a systems-level understanding of aging and cancer: an interview with Dirk Bohmann

**DOI:** 10.1242/dmm.016329

**Published:** 2014-05

## Abstract

The Bohmann lab, based at the University of Rochester Medical Center in New York, uses *Drosophila* to explore the cross-talk between stress and metabolic regulation, with the goal of understanding how the signaling pathways involved can drive aging, cancer and other degenerative pathologies. In this interview, Dirk Bohmann describes his motives for moving into the fly field, explains how the study of aging can give important insights into complex disease mechanisms, and advocates using a systems-biology approach to advance drug discovery.

Dirk Bohmann was born in Germany in 1960. He earned a PhD in Biology from the University of Tübingen in 1986, gaining insights into eukaryotic gene regulation in the lab of Walter Keller. He then joined Robert Tjian’s group at the University of California at Berkeley as a post-doctoral fellow. At Berkeley, Dirk continued to explore transcription factor biochemistry and regulation, and it was during this time that he conducted breakthrough work that identified c-Jun, a proto-oncogene product, as a gene-specific transcriptional regulator. Moving back to Germany in 1989, he set up a research group at the European Molecular Biology Laboratory (EMBL) in Heidelberg, and continued to study the function and regulation of transcription factors. Shortly after arriving at the EMBL, he adopted the fruit fly system to explore the biology regulated by these transcription factors. *Drosophila* genetics has remained at the forefront of his research methodology since this time. In 2001, Dirk took on a faculty position in the Department of Biomedical Genetics at the University of Rochester Medical Center, NY. Over the past 20+ years, his lab has made important contributions to our understanding of the JNK and Nrf2 signaling pathways (among others), including their involvement in development and malignancy. The group is also interested in the role of oxidative stress and metabolic regulation in aging and disease, and has used *Drosophila* models to define crucial underlying mechanisms. In this interview, he discusses how a multifaceted systems-biology approach can be used to guide therapy development for cancer and age-related pathologies. He currently holds a professorship at the University of Rochester, and also recently joined the team of academic editors at *Disease Models & Mechanisms*.

**How and when did you become interested in science?**

My father has a PhD in Chemistry and his uncle was a chemist as well, so there’s a longstanding scientific tradition in the family. I’ve always been interested in biology at some level. An important experience for me was being taught in high school by some really inspiring biology teachers – that’s definitely where I caught the bug. High school education is really important for shaping young people’s interest in science and getting them started on that road. I think this is something that the scientific research community, journals and organizations that communicate scientific discoveries should take more seriously. Some institutions like the National Science Foundation (NSF) and the Howard Hughes Medical Institute (HHMI) are already promoting science education in high schools, but even as individual scientists we should be more mindful of the importance of reaching out to the community and especially to young people.

**Figure:**
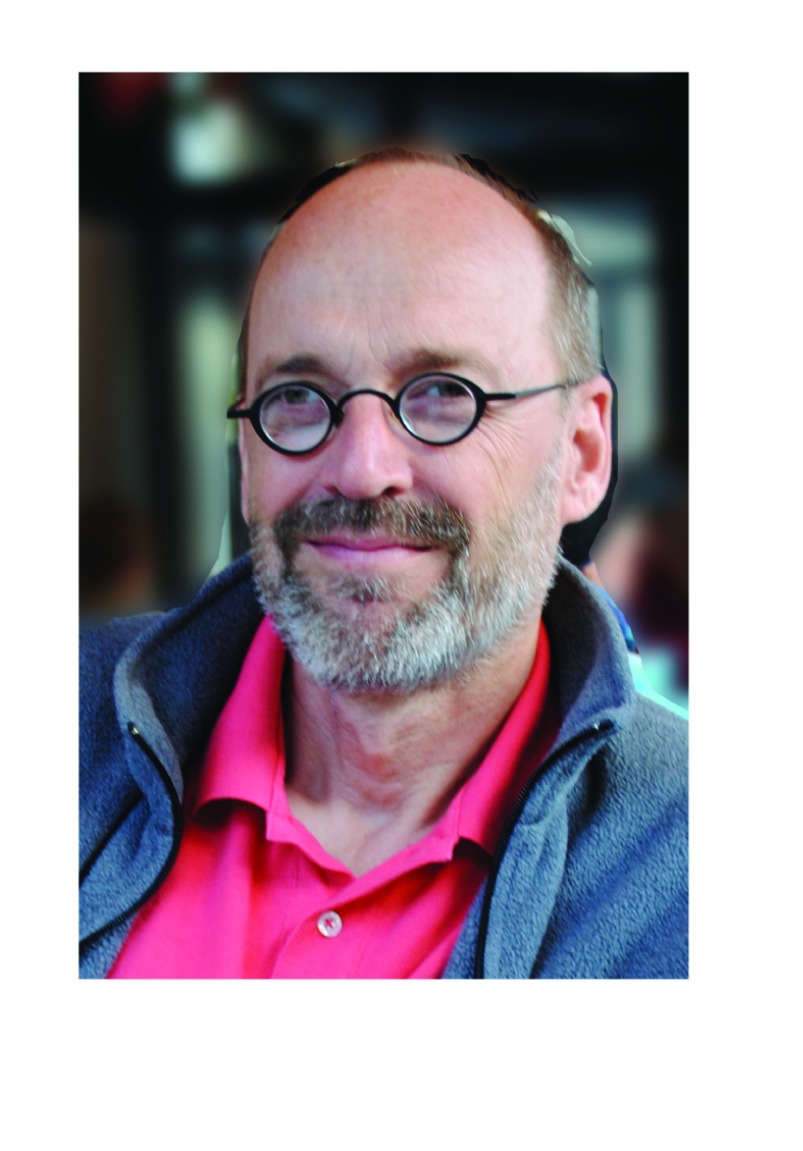


**What do you think could be done to promote science among younger students?**

I recently read the biography of Werner Heisenberg [German theoretical physicist], who started his career almost 100 years ago. In those days there were no graduate schools in today’s sense and a talented young student like Heisenberg could work and interact directly with people like Niels Bohr and Max Born, straight out of high school. At that time, particularly in physics, a lot of Nobel prizes were awarded based on work the individual did before they were 30. Nowadays, the framework of scientific education is more formalized and rigid and it’s typically not until the late 20s that a budding scientist starts to be immediately involved in scientific discovery and discussion. We are missing out on a potentially very productive phase when young people could be incredibly creative and brilliant; instead they spend their time in classrooms or lecture halls. Perhaps related to this, there appears to be less interest, particularly among American students, in science than we would like. A lot of high school graduates are more attracted to business, law or medical careers. I think that engaging kids in science early on, in different and creative ways, would promote more interest in the subject in the younger generation. This isn’t something that can be done by one group – scientists, teachers or journalists – alone. For example, TV and media could be a powerful force to promote the excitement of science.

**Where and with whom did you train after high school?**

I did my PhD research at the German Cancer Research Center in Heidelberg with Walter Keller, who is a biochemist. At the time, the Keller lab worked on eukaryotic transcription and RNA processing. This was in the early-to-mid ’80s, and it was an exciting time because we were starting to understand the principles of eukaryotic gene regulation at a molecular level. I interacted with a lot of researchers, including Iain Mattaj, Walter Schaffner, Günther Schütz, Peter Gruss and Hans Schöler, who were all heavily involved in this kind of ‘gold rush’ stage. I then moved to the University of California in Berkeley, to take a post-doc position in Robert Tjian’s lab. There, I isolated transcription factors that regulate the genes of the DNA tumor virus SV40, then a preeminent model for eukaryotic gene regulation. It was very exciting to discover that one of these factors was encoded by a proto-oncogene, called *c-Jun*, that had been isolated by Peter Vogt a few months earlier, based on studies on retroviruses. The identification of c-Jun as a DNA-binding transcription factor suddenly opened a whole new research area for me. One day I was working on a rather arcane model of eukaryotic transcription regulation, and the next day I found myself at the center of the cancer and growth-control research. That unexpected leap, to me, is what makes science so thrilling.

**What is the major research focus of your lab?**

I moved from Berkeley back to Germany and took on a group-leader position at the EMBL in Heidelberg, and, in the early days, my work remained centered on the biochemistry of transcription factors. It soon became clear to me that the next interesting question was to understand how these biochemical functions affected biological processes at the cell or organism level. In order to address this question, I needed a genetic system. So, I started doing some experiments on the side using *Drosophila*, with my friend Marek Mlodzik, who worked in the lab next door, and whom I had known since the Berkeley days. We started a very fruitful collaboration on the function of transcription factors in signal-transduction pathways that control *Drosophila* development. This opened up a whole new dimension of scientific enquiry to me, and from these beginnings *Drosophila* became more and more prevalent in the scientific work of my lab.

Over the years, we have used both mammalian cell culture and fly systems to investigate how signal-transduction and gene-regulation pathways control biology and disease. In the recent past, we have concentrated primarily on understanding the systems that help the cell to deal with stressful insults. We would like to understand mechanisms underlying aging and age-associated diseases, including cancer and neurodegeneration. Currently, I am interested in the role of stress and redox regulation in the aging organism: how systems that maintain homeostasis fall apart as we age, and what could be done to preserve or even restore their function. This might feed into more disease-centered projects: aging research has already advanced our understanding and ability to treat metabolic syndrome and related diseases. Understanding aging more completely could help us to come up with preventative strategies that may delay the onset of these and other pathologies that are prevalent in the aging population.

**Could the study of aging guide therapy development for complex diseases?**

Although aging isn’t a disease itself, it’s probably the biggest risk factor for almost any disease. It is known that aging and lifespan are influenced by genetic factors, given the different lifespans of various organisms, or the fact that longevity can run in families. Some exciting discoveries on the nature of genetic regulators of aging have emerged from studying model organisms over the last 10 to 20 years, particularly from work by Cynthia Kenyon [at UCSF], Leonard Guarente [MIT] and Gary Ruvkun [Harvard]. Their studies first confirmed that aging is genetically determined by specific molecular signals, but they also showed how aging can be regulated in response to external conditions. While we’ve known for almost a century that calorie restriction, a reduction of food intake without malnutrition, can increase life span, we’re now starting to understand the pathways involved. Inspiring studies in *C. elegans* and *Drosophila* have shown that these are open to interference and manipulation that can extend lifespan and ‘healthspan’, the duration of relative health and fitness in an organism’s life. These studies suggest that the pathways that mediate lifespan extension in response to pharmacological manipulations might be separate from other effects of caloric restriction, including extreme weight loss, and decreased fertility and libido, which are certainly side effects that most of us wouldn’t be very happy with. A number of biotech companies are actively trying to utilize this knowledge for therapeutic benefit.

Whether it’s aging, cancer, Alzheimer’s disease or diabetes, we should approach these complex processes from the perspective of a biologist rather than a pharmacologist. We’re used to visualizing malfunctions of linear signaling pathways as a cause for many maladies. This gives rise to the impression that all we have to do to cure a disease is eliminate or activate one particular component in a pathway – this is, in most cases, a gross over-simplification. Although a lot of successful therapies have been developed by homing in on specific targets and then identifying a drug to manipulate its function, there’s even greater potential for developing cures if we achieve a deeper understanding of underlying disease mechanisms. For example, in progressive conditions such as cancer or neurodegeneration, regulators may have different functions at early and late stages of the disease. Therapies may therefore change in their efficacy. We also need to consider regulating multiple targets in specific organ systems, or systemically active endocrine targets. Approaching disease research in a holistic, or ‘systems biology’, way will give us a more comprehensive view of how multiple factors contribute to pathology. Such a view would facilitate the development of smarter, targeted therapies. An increasing appreciation of this approach is the reason that medical schools like the University of Rochester hire ‘fly guys’ like me – to provide a systems-level view of universal biological mechanisms and of the way in which these mechanisms can go awry. Disease models, whether organism-based, cellular or computational, are an important aspect of this approach and journals such as DMM help to promote it.

**What makes *Drosophila* such a great model for understanding complex biological processes in humans?**

*Drosophila* is simple, easy and cheap to work with, and has a short generation time. In many ways, it is amazingly similar to us, which wasn’t at all obvious when fly research started about 100 years ago at Columbia University with Thomas Morgan. It wasn’t even obvious 30–40 years ago, before the first genes that are affected in developmental mutants were identified. It is now clear that in many fundamental ways the genes and mechanisms that control the function and development of the insect body are preserved in humans. Thus, early studies on the genetic basis of fly development led to a treasure trove of information that still benefits biomedical research. The ease of experimentation, the relative simplicity of the organism and its genome, and the relative lack of genetic redundancy (which facilitates unbiased genetic screening), are all major strengths. Also, a large number of very sophisticated methods for gene modulation and imaging are available.

One other thing that I particularly enjoy is the collegiality of the fly field. People are very open, and they have built invaluable resources for the community, such as FlyBase, an immense web resource that collects and interrelates genetic, genomic and phenotypic information, or stock centers. In general, there is a lot of collaboration and sharing. This makes working on flies productive and enjoyable. Finally – the thing I’m often reminded of at the dinner table, because my wife works with mice – fly research is free from a lot of the regulatory impediments that govern research using mice and other vertebrates.

On the other hand, we have to be aware of the limitations. Particularly within the medical school, we should always question the medical and societal benefit of the work. While we can clearly generate an important knowledge base in *Drosophila*, ultimately, we have to move this into a more translational arena by collaborating with people who work with vertebrate models or humans. In our department, we try to bring people who work with different technologies and systems together, to help them to understand that they actually have common goals. The utility and the power of studying model organisms will grow as we learn to move from one model to another with increasing ease. I don’t see myself exclusively as a fly guy; I keep in mind that ultimately the goal of life-science research is the discovery of universal truths and answers to biological questions that transcend a specific model organism. This is something I also try to teach to our students.

**What other advice do you give to young scientists hoping to follow in your footsteps?**

I have Robert Tjian at Berkeley to thank for teaching me a certain style of science. He told me that if you had an idea, you had to persevere and really have a certain “stick with it-ness”. I try to convey this to my students. Also, I emphasize the importance of scientific communication. You can’t be a brilliant scientist if you can’t explain your ideas and your results to others. I encourage students in my lab to be open-minded. For example, research grants normally cover 5 years and the research during this period is guided by the experimental plan laid out at the beginning. But one should not stick to such a plan at all costs. The most interesting things that are discovered are often those that we don’t foresee. I think it is very important to recognize an interesting result that might point you in a direction that you hadn’t anticipated. These types of moments are what really make it worth going forward.

**What’s been your most exciting research finding to date?**

I would be kind of sad if there was only one. It’s always the next big thing that you think is around the corner that’s most exciting at any given time. In terms of broad impact, it is probably the discovery I made as a post-doc in Berkeley. I discovered the biochemical function of the c-Jun oncoprotein and showed that it’s a component of the AP1 transcription factor. Since then my lab has made a number of discoveries that have advanced our understanding and have fuelled the work of the lab. We have implicated a number of genes and regulators in the control of aging, and gained insight into the interactions between stress and insulin signaling. We’ve done some exciting work on the control of transcription factor activity by ubiquitylation, and on the role of stress signaling pathways in cell migration during development and in cancer. There is always something to be excited about, and I’m much more interested in looking forward to the next thing than looking backward at past glories.

**What would you say are the most urgent challenges in the cancer field?**

One thing that is on everybody’s mind in cancer research is the merging of genomic technology and cancer treatment to provide personalized medicine. Of course, the increasing use of whole-genome sequencing for cancer diagnosis and personalized therapy poses all sorts of challenges in terms of data handling, data privacy and implementation in a day-to-day clinical setting. From the basic-science standpoint, success in this venture is something that I think will be driven by gaining a better biological understanding of the cellular and molecular origins of cancer. For this, we need to focus on the complexities and emergent properties of the tumor cell, compared to healthy cells. This type of analysis will lead to the identification of a cancer cell’s Achilles heel. Exploiting such defined vulnerabilities is the basis for the development of rational therapies. Clearly, disease models will be central in this endeavor.

An area that is rapidly developing and will be important is the interplay between metabolic regulation and cancer. Researchers are starting to appreciate that metabolic changes are crucial in the transformation of a normal cell to a cancer cell. Understanding these changes might tell us something about cancer cell characteristics. Biochemical processes we thought we could safely forget after biochemistry class in school, such as the Krebs cycle and glycolysis, have thus become very relevant again. As for many research questions, a multi-pronged, multidisciplinary approach might give us new and unexpected insights that could lead to original therapeutic modalities.

Another aspect of cancer biology that is interesting but poorly understood relates to the similarities between stem cells and cancer cells. In our lab, we’re interested in the role of redox signaling. We know that cells change the way in which they control their redox status when they go from a stem cell to a differentiated cell, and that cancer cells somehow acquire some stem-cell-like properties. We still have an incomplete understanding of these changes, but the metabolism and redox balance of cancer cells deserves a lot more scrutiny.

As I’ve touched upon, we should look at multiple target points for treating cancer instead of pinpointing single targets. Again, this is where model systems can make a valuable contribution. For example, Ross Cagan [Professor at Mount Sinai School of Medicine, and DMM Editor-in-Chief] has shown very beautifully that you can use fly genetics to understand the complex interplay of multiple drug targets in the control of cancer progression in an organism. Such approaches are amazingly translatable. His work provides a good example of how understanding cancer and other progressive degenerative diseases can be advanced using a biological approach rather than the classical single-drug-target pharmacological view.

**How do you relax and have fun away from the lab?**

I like to cycle, both commuting and in my free time, and my wife and I ride about 2000 miles a year. We have fun spending time with our teenage kids. My daughter is an active rower and my son plays basketball, and we enjoy watching them compete. We also like to travel together.

